# The role and mechanisms of gram-negative bacterial outer membrane vesicles in inflammatory diseases

**DOI:** 10.3389/fimmu.2023.1157813

**Published:** 2023-06-16

**Authors:** Shuoling Chen, Qian Lei, Xianghui Zou, Dandan Ma

**Affiliations:** Department of Endodontics, Stomatological Hospital, School of Stomatology, Southern Medical University, Guangzhou, China

**Keywords:** outer membrane vesicles, inflammatory diseases, periodontal disease, gut microbiota, pattern recognition receptors, inflammasomes, atherosclerosis, Alzheimer’s disease

## Abstract

Outer membrane vesicles (OMVs) are spherical, bilayered, and nanosized membrane vesicles that are secreted from gram-negative bacteria. OMVs play a pivotal role in delivering lipopolysaccharide, proteins and other virulence factors to target cells. Multiple studies have found that OMVs participate in various inflammatory diseases, including periodontal disease, gastrointestinal inflammation, pulmonary inflammation and sepsis, by triggering pattern recognition receptors, activating inflammasomes and inducing mitochondrial dysfunction. OMVs also affect inflammation in distant organs or tissues via long-distance cargo transport in various diseases, including atherosclerosis and Alzheimer’s disease. In this review, we primarily summarize the role of OMVs in inflammatory diseases, describe the mechanism through which OMVs participate in inflammatory signal cascades, and discuss the effects of OMVs on pathogenic processes in distant organs or tissues with the aim of providing novel insights into the role and mechanism of OMVs in inflammatory diseases and the prevention and treatment of OMV-mediated inflammatory diseases.

## 1 Introduction

All gram-negative bacteria secrete outer membrane vesicles (OMVs) during planktonic growth and in surface-attached biofilm communities (1). These spherical, nanosized membrane vesicles are released from the outer cell membrane, and they consist of lipopolysaccharide (LPS), peptidoglycan (PG), proteins, DNA, RNA, and other virulence factors such as enzymes and toxins (1, 2). Compared to their parent bacteria, OMVs are nanosized and contain a relatively high concentration of toxins and virulence factors (3). During infection, pathogenic factors are exposed to host cells via these vesicles, and OMVs can bypass direct contact between cells. Therefore, OMVs can establish bacteria-host communication and initiate pathogenesis in the absence of living bacteria (4).

In recent years, studies have connected OMVs with inflammatory disease processes. The type of inflammatory diseases include chronic inflammatory diseases, autoimmune diseases, and inflammation-related tumors. The role of OMVs in inflammatory diseases, particularly periodontal disease, gastrointestinal inflammation (including inflammatory bowel disease), pulmonary inflammation, sepsis and inflammation-related tumors, has been reported extensively. For example, *Fusobacterium nucleatum* (*F. nucleatum*) OMVs increased the number of osteoclasts and increased the production of inflammatory cytokines in gingival connective tissues, leading to more severe periodontitis symptoms (5). *Helicobacter pylori* (*H. pylori*) OMVs containing virulence factors were rapidly internalized by gastric epithelial cells and promoted the destruction of the mucin barrier and bacterial colonization in gastric disease development (1). OMVs derived from *Porphyromonas gingivalis* (*P. gingivalis*) significantly disrupted the tight junction proteins among lung epithelial cells and exerted cytotoxic effects leading to pulmonary inflammation (6, 7). *Escherichia coli* (*E. coli*) OMVs have been shown to increase the expression of IL-6, P-selectin, and intercellular adhesion molecules and markedly decreased the expression of thrombomodulin, ultimately leading to the coagulation cascade (8). Small RNA-23392 (sRNA-23392) packaged by *P. gingivalis* OMVs promoted oral squamous cell carcinoma cell migration and invasion by targeting desmocollin-2 (9).

Generally, OMVs participate in inflammatory diseases initiated by Toll-like receptor (TLR) signaling and nucleotide binding oligomerization (NOD)-like receptor (NLR) signaling. LPS is recognized by TLR4, PG by TLR2, NOD1 and NOD2, and lipopeptides/proteins are recognized by TLR2, DNA by TLR9 (10–13). New insights on the interactions between OMVs and target cells have been developed in the last 5 years. The outer membrane proteins FomA and OmpA have recently been reported to be recognized by TLR2, flagellin by TLR5 and RNA by TLR7 and TLR8 (14–17). Furthermore, OMV activation of inflammasomes and mitochondrial dysfunction can induce inflammation (18, 19). Recent research has revealed that OMVs contribute to inflammation by mediating cell death (20).

OMVs play a role in long-distance cargo transport and have been implicated in distant organ or tissue inflammation, particularly in atherosclerosis (AS) and Alzheimer’s disease (AD). A study of OMVs from CagA-positive *H. pylori* demonstrated that these factors significantly increased cholesterol levels, promoted apoptosis in the arterial lumen and accelerated coronary artery atherosclerotic plaque formation in ApoE-/- mice, an animal model of spontaneous atherosclerosis (21). OMVs from the periodontal pathogen *P. gingivalis* were involved in activating glial cells, and ultimately stimulating neuroinflammation and memory dysfunction (22). Therefore, determining the role of OMVs from different sources in distant organ or tissue inflammation would be beneficial.

To date, only the review by Yu et al. (2018) has addressed the role of OMVs in inflammatory diseases (such as pulmonary inflammation and sepsis), and they mainly summarized the effects of gram-negative and gram-positive bacterial membrane vesicles on various mammalian cells (23). In the present review, we focused on the role of OMVs in inflammatory diseases, examine the mechanism by which OMVs participate in inflammatory cascades, and discuss the effects of OMVs on the inflammatory pathology of distant organs or tissues with the aim of providing novel ideas for the mechanism of OMVs in inflammatory diseases and the prevention and treatment of OMV-mediated inflammatory diseases.

## 2 Characteristics of OMVs

Bacterial OMVs are secreted by gram-negative bacteria into their surroundings and are approximately 20 to 350 nm in size (1). Originally identified as a product of the cell wall, OMVs are now considered to be part of a general secretory system (24, 25). The secretion of OMVs is an omnipresent process and occurs in numerous bacteria, including *P. gingivalis*, *H. pylori*, enterohemorrhagic *E. coli* (EHEC), *F. nucleatum*, *Pseudomonas aeruginosa* (*P. aeruginosa*), and *E. coli* Nissl 1917 (EcN) (26–29). Recently, reports have shed light on OMV biogenesis, which is based on the PG layer having a low amount of lipoproteins, the presence of PG residues with autolysins, an increase in negatively charged LPS within the cell envelope, and phospholipid accumulation (30).

Due to the mode of formation, OMVs have been shown to harbor various contents of the parental bacteria. OMVs are composed of many inflammatory substances, such as LPS, phospholipids, PG, outer membrane proteins, DNA, RNA, and other virulence factors such as enzymes and toxins (31). These vesicles also contain anti-inflammatory factors that play a role in limiting the immune response, such as polysaccharide and sphingolipid (32, 33). However, OMVs produced by different bacteria and different strains of a bacterial species vary in species and cargo quantity. The mechanism of enriching factors is not fully understood and need to be further investigated in depth.

High concentrations of cargoes can be protected from degradation due to the membrane structure. The cargoes in OMVs can perform specialized functions in bacteria-bacteria interactions and bacteria-host interactions. The defensive functions include the enhancement of bacterial survival by communication through quorum sensing, stress response adaptation, nutrient acquisition, antibiotic resistance and biofilm formation (34–38). Their offensive functions play vital roles in pathogenic processes, such as the transmission of toxins and virulence factors into target cells, the establishment of a colonization niche, and the modulation of host inflammation and the immune response (39, 40). These characteristics determine the wide roles of OMVs in various inflammatory diseases. Next, we will elaborate in detail.

## 3 The role of OMVs in inflammatory diseases

### 3.1 OMVs in periodontal disease

Periodontal disease is a kind of inflammatory disease induced by the interaction between aberrant immune responses and dysbiosis of microbial communities within periodontal tissues (41). It is classically characterized by gingival bleeding, alveolar bone absorption, attachment loss, periodontal pocket formation, and even tooth loss (3).

#### 3.1.1 OMVs affect biofilm formation and bacterial invasion

**Figure 1:**
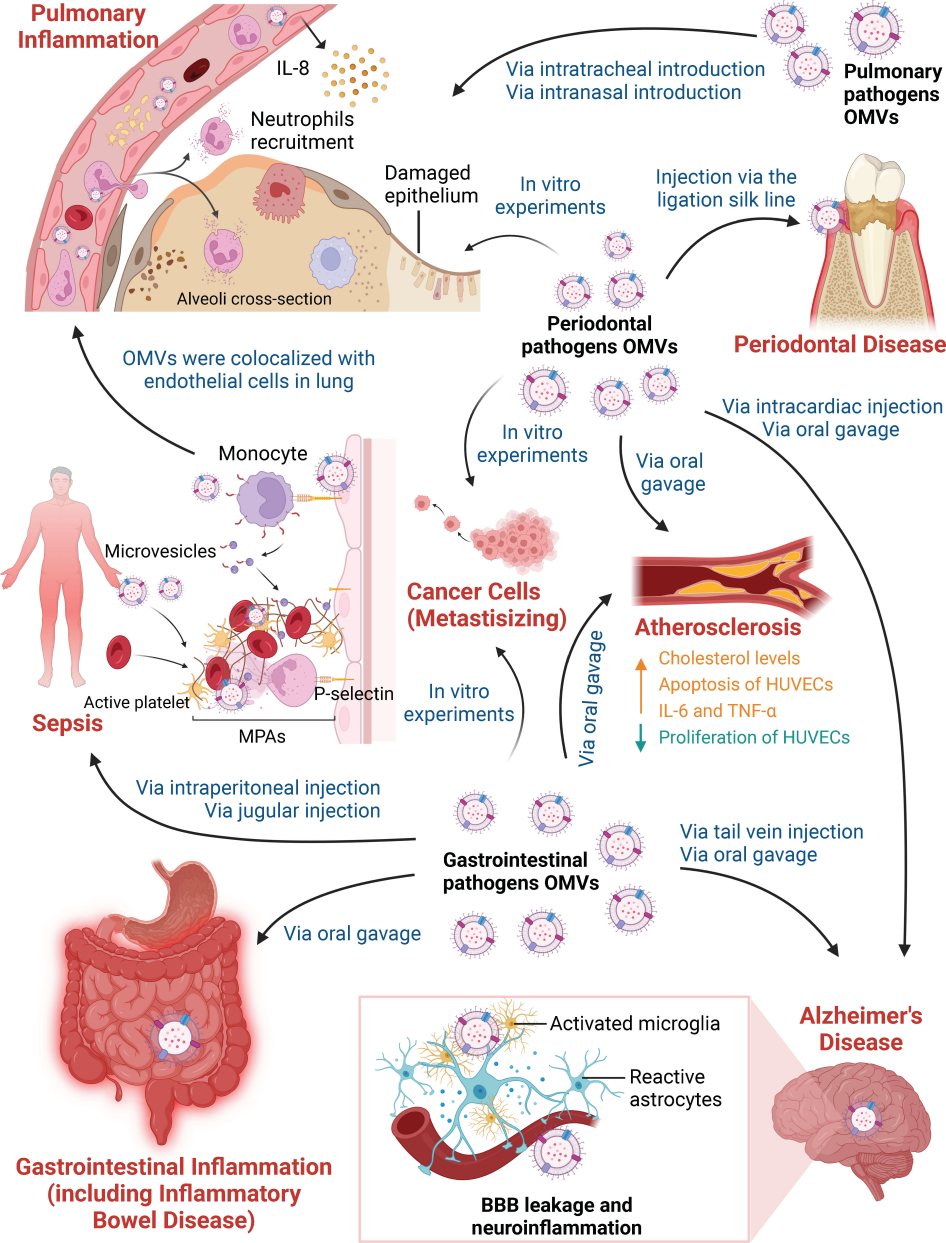
Different Pathways by which Outer Membrane Vesicles Participate in Various Inflammatory Diseases. OMVs derived from periodontal pathogens, gastrointestinal pathogens and pulmonary pathogens participate in inflammatory diseases and affect distant organ or tissue inflammation in the absence of living bacteria. On the one hand, *in vivo* and *in vitro* experiments have focused on OMVs in periodontal disease, gastrointestinal inflammation, pulmonary inflammation, sepsis and inflammatory-related tumors. Periodontal pathogen OMVs have been shown to induce local chronic inflammation and bone resorption in periodontal tissue after being injected via the ligation silk line. OMVs can kill lung epithelial cells due to cytotoxic effects *in vitro*. Gastrointestinal pathogen OMVs promote the destruction of the gastrointestinal mucin barrier and the production of inflammatory cytokines when administered by oral gavage. OMVs contribute to a hyperactive inflammatory system after intraperitoneal injection and jugular injection. Stimulation of HUVECs promotes the activation of platelets and the formation of prothrombotic MPAs, thus increasing thrombus formation in sepsis. OMVs can spread and accumulate in the lung, resulting in neutrophil recruitment and IL-8 production by vascular endothelial cells. OMVs derived from pulmonary pathogens also directly trigger pulmonary inflammation. In addition, periodontal pathogen OMVs and gastrointestinal pathogen OMVs can promote the invasion and migration of cancer cell line *in vitro*. On the other hand, periodontal pathogen and gastrointestinal pathogen OMVs play vital roles in AS and AD based on long-distance cargo transport. After oral gavage, OMVs lead to high cholesterol levels and the release of IL-6 and TNF-α, as well as the suppression of proliferation and the promotion of apoptosis in HUVECs in AS. In addition, periodontal pathogen OMVs deliver cargoes that cross the blood-brain barrier (BBB) after intracardiac injection or oral gavage. Gastrointestinal pathogen OMVs increase BBB permeability after tail vein injection. OMVs degrade tight junction proteins and activate microglia and astrocytes, which are responsible for BBB leakage and neuroinflammation in AD.

Numerous studies have reported the clinical importance of OMVs in plaque biofilm formation by oral microorganism coaggregation and the invasion of oral epithelial cells during the pathogenesis of periodontal disease; similar to their parent bacteria (**Figure 1**). *P. gingivalis* OMVs could promote coaggregation between mycelium-type *Candida albicans* and *Staphylococcus aureus* and between *Treponema denticola* (*T. denticola*) and *Lachnoanaerobaculum saburreum* (42). OMVs also increased the invasion and adhesion of *Tannerella forsythia* (*T. forsythia*) to oral epithelial cells (43). However, proteases of *P. gingivalis* OMVs decreased the auto-aggregation of *F. nucleatum*, and *P. gingivalis* OMVs inhibited the invasion of *F. nucleatum* by downregulating FadA and FomA (43). A comparative study revealed significantly higher invasive capability by *P. gingivalis* OMVs than *P. gingivalis* itself (6). It is well documented that *P. gingivalis* mediates invasion through the interaction between the fimbriae and receptors on host cells (44). However, the mechanism underlying the invasion of *P. gingivalis* OMVs remains unclear. Fimbriae have not yet been observed on OMVs. Several well-known adhesins, such as gingipains and fimbrial proteins, are considered potential weapons (45). Surprisingly, sphingolipid-containing *P. gingivalis* OMVs can serve as a delivery vehicle to limit the macrophage immune response to *P. gingivalis* (33). The sphingolipid-null *P. gingivalis* OMVs induce a hyperinflammatory immune response with increased levels of TNF-α, IL-1b, and IL-6 (33). These findings suggest that *P. gingivalis* OMVs induce pathogen recognition and/or inflammation response in a manner that can be limited by sphingolipids.

#### 3.1.2 OMVs induce inflammation in periodontal tissue

OMVs were shown to dysregulate the immune response and induce local chronic inflammation in periodontal tissue (**Figure 1**). *P. gingivalis* OMVs have been shown to promote the infiltration of neutrophils in connective tissue, stimulate glycolysis, increase mitochondrial reactive oxygen species (ROS) production and induce macrophages to produce large amounts of IL-6, IL-12p70, TNF-α, IFN-β and nitric oxide (46). OMVs from *P. gingivalis* and *T. forsythia* stimulated TLR2 signaling via lipoproteins and/or LPS and induced the production of osteoclastogenic cytokines, such as IL-6, IL-1β, and TNF-α, leading to alveolar bone loss (47). However, it remains unclear whether or how OMVs affect osteoblasts in periodontal disease. Further studies are warranted to examine the role of bacterial OMVs in osteoblast differentiation, proliferation and mineralization.

In an animal model of periodontitis, after being activated by *F. nucleatum* OMVs, mouse gingival fibroblast apoptosis and lactate dehydrogenase release were promoted, and M0 macrophages transformed into M1 macrophages, which produced more inflammatory cytokines (5). The periodontitis symptoms of mice treated with OMVs were more serious, and there were more osteoclasts and more inflammatory factors in their gingival tissues (5). However, it is not clear which OMV cargoes are involved in periodontal disease. Since FadA in *F. nucleatum* was reported to induce bone loss, scientists might reveal whether their OMVs enhance the development of periodontitis by knocking out the expression of FadA in *F. nucleatum* (48).

### 3.2 OMVs in gastrointestinal inflammation

The gastrointestinal tract is a complex ecosystem that maintains homeostasis through interactions between the barrier system and microorganisms. The highly compacted inner mucin layer effectively prevents bacteria and their products from accessing epithelial cells (49). Bacterial OMVs can deliver various bacterial components to epithelial cells across the mucin layer, which is physically inaccessible to bacteria (50–52) (**Figure 1**).

#### 3.2.1 OMVs mediate proinflammatory effects in gastric inflammation

Accordingly, *H. pylori* OMVs transport several virulence factors, such as cytotoxin-associated gene A (CagA), vacuolating cytotoxin A (VacA) and catalase (KatA) (53). CagA can disrupt the tight junctions of the epithelium, VacA can induce vacuolization, and high concentrations of KatA exerts a strong antioxidant effect that protects the bacteria against oxidative damage induced by the immune response (1). OMVs are internalized by the gastric epithelium and then promote the destruction of the mucin barrier and bacterial colonization. In addition, the effect of *H. pylori* OMVs is dose dependent, affects epithelial cell proliferation and induces the release of IL-6, TNF-α, and IL-8 (54). At low doses, OMVs increase AGS cell (human gastric adenocarcinoma cell line) proliferation, while at high doses, growth arrest, a decrease in proliferation, an increase in toxicity and IL-8 production are observed (55). Thus, OMVs can promote low-grade gastritis associated with *H. pylori* bacterial infection (55). It should be noted that *H. pylori*-derived OMVs from hosts with various gastric diseases have different effects on inducing inflammation (56). Further studies are warranted to differentiate the expression pattern of pathogenic cargoes among *H. pylori* OMVs associated with various gastric diseases.

#### 3.2.2 OMVs mediate inflammatory and anti-inflammatory effects in intestinal inflammation

The proinflammatory and immunomodulatory effects of intestinal bacterial OMVs also have pathogenetic implications. EHEC O157 OMVs containing LPS and flagellin induced IL-8 production in human intestinal epithelial cells (IECs) by activating TLR signaling and NF-κB signaling (16). OMV-associated virulence factors are internalized by Caco-2 cells and human brain microvascular endothelial cells via dynamin-dependent endocytosis and trigger target cell apoptosis, which may contribute to diarrhea and hemolytic uremic syndrome (57). In addition, *Vibrio cholerae* (*V. cholerae*) OMVs modulate the epithelial proinflammatory response via the translocation of *V. cholerae* cytolysin (58). Activated dendritic cells stimulated with these OMVs promoted T-cell polarization toward an inflammatory Th2/Th17 response (58, 59). Proinflammatory factors are also present in IECs treated with *F. nucleatum* OMVs and *Campylobacter jejuni* OMVs (60, 61). Furthermore, previous reports have highlighted that intestinal commensal bacterial OMVs could mediate anti-inflammatory and barrier protection effects (62). For instance, colitis mice treated with EcN OMVs exhibited a reversal of the dextran sodium sulfate (DSS)-induced reduction in the anti-inflammatory cytokine IL-10 and a decrease in proinflammatory cytokines, such as TNF-α, IL-1β, and IL-17 (63). A previous study indicated that *Bacteroides fragilis* (*B. fragilis*) release OMVs with immunomodulatory and protective effects against colitis (32). Dendritic cells are able to sense *B. fragilis* OMV-associated polysaccharide through TLR2, resulting in enhanced regulatory T cells and anti-inflammatory cytokine production (32). *Bacteroides vulgatus* OMVs have been shown to induce tolerant semimature dendritic cells for immunomodulation and the maintenance of a balanced gut microbiota (64). OMVs derived from *Odoribacter splanchnicus*, *Akkermansia muciniphila* (*A. muciniphila*) and *Faecalibacterium prausnitzii* also downregulate proinflammatory cytokines (62, 65–67). These studies fully demonstrate the immunoregulatory effects of gut microbiota OMVs and provide ideas for the development of therapeutic strategies targeting the effects of OMVs. However, most studies lack in-depth exploration of the mechanisms of pathogenic and anti-inflammatory substances. The presence, amount and functions of these substances associated with OMVs depend greatly on the bacterial strain. Therefore, further research is needed to explore the role of the individual virulence factors within OMVs in gastrointestinal inflammation.

#### 3.2.3 OMVs mediate immune modulation in inflammatory bowel disease

Under physiological conditions, the gut microbiota interacts with the host and maintains intestinal immune homeostasis, which has been implicated in the maturation and functions of intestinal epithelial cells and immune cells. Dysbiosis of the gut microbiota can effect intestinal homeostasis by OMVs mediating cross-talk between microbiota and intestinal immunity. Increasing studies revealed that OMVs mediate immune modulation in inflammatory bowel disease (IBD), a type of complex autoimmune disease associated with genetic susceptibility, dysbiosis of the gut microbiota and immune system imbalance. *F. nucleatum* OMVs compromise intestinal barrier by significantly promoting the differentiation of proinflammatory macrophages and oxidative stress damage and accelerating RIPK1-mediated intestinal epithelial cells necroptosis (68). In the DSS-induced colitis animal model, *A. muciniphila* OMVs can ameliorate intestinal inflammation and also protect against colitis phenotypes, such as weight loss, colon length, and inflammatory cell infiltration of colon wall (67). In addition, *Bacteroides thetaiotaomicron* (*B. thetaiotaomicron*) OMVs stimulate significant IL-10 expression by colonic dendritic cells and significant IL-6 expression by peripheral blood-derived dendritic cells in healthy individuals (69). However, there are reduced numbers of regulatory dendritic cells in the colon and lower proportion of dendritic cells that express IL-10 in the blood of IBD patients (69). This finding suggests that IBD patients may show disturbance of the immune response balance directed by *B. thetaiotaomicron* OMVs. These studies provide novel insights into the role of OMVs in IBD and encourage us to focus on the functions of pathogenic bacteria and probiotics in health or disease conditions. It should be noted that the available evidence supporting the use of bacterial OMVs as a remedy for IBD is comparatively scarce. More mechanisms of the interactions mediated by OMVs between the intestinal microenvironment and the host need to be identified and applied clinically.

However, except IBD, the role of OMVs in other autoimmune diseases has not been reported. Recent study indicated that exosomes of OMV-stimulated macrophages are found to contain OMV-derived proteins (70). These exosomes may carry autoantigens and stimulate B cells to generate autoantibodies (71). In addition, exosomes may carry complement components or serve as a platform to activate complement system (71). These findings expand our understanding of potential mechanisms of OMVs in autoimmune diseases, but the further in-depth study is still needed.

### 3.3 OMVs in pulmonary inflammation

The epithelial cell layer of the surface of airways and alveoli provides the primary defense against microorganism invasion (72). Once invasion impairs the lung epithelial barrier, a series of pathologies occur, such as inflammation and fibrosis (7). It is known that microorganisms employ OMVs to invade the lung epithelial barrier without making direct contact with host cells, mediating inflammatory responses *in vitro* and *in vivo* (**Figure 1**).

#### 3.3.1 OMVs cause pulmonary inflammation in animal models

OMVs administered via the trachea, nasal cavity and enterocoelia have been shown to induce pulmonary inflammation in animals (**Figure 1**). Intratracheal administration of *Acinetobacter nosocomialis*-derived and *Stenotrophomonas maltophilia*-derived OMVs could stimulate high expression of proinflammatory cytokines and chemokines and induce early inflammatory responses such as congestion and focal neutrophilic infiltration *in vivo* (73, 74). Intranasal administration of OMVs secreted by *P. aeruginosa*, *Moraxella catarrhalis* (*M. catarrhalis*), and *Acinetobacter baumannii* (*A. baumannii*) resulted in pulmonary inflammation (75–78). The response was even greater than the induction of cytokines by purified LPS (76). Weight loss in an *A. baumannii* OMV-induced experiment indicated the potential relationship between OMVs and metabolism regulation (78). Another study showed that intraperitoneally injected *E. coli* OMVs could spread and accumulate in the lung and thereby induce systemic inflammatory response syndrome (SIRS), characterized by systemic and pulmonary inflammation (79). Increased levels of IL-6 and TNF-α were observed. Mechanistically, these factors recruited neutrophils to the lung via IL-8/CXCL1 released from vascular endothelial cells in TLR4- and NF-κB-dependent manners, leading to a strong pulmonary inflammatory response (79). This was the first report regarding OMV-induced production of IL-8 by vascular endothelial cells. Specific pathogenic components and the mechanisms of bacterial OMV involvement in pulmonary inflammation need to be examined. Intriguingly, *P. aeruginosa* OMVs not only induced the host innate immune response via the MAPK signaling pathway but also delivered specific bacterial sRNA-52320 to resist the innate immune response (80). sRNA-52320 subsequently suppressed the inflammatory response mediated by the MAPK signaling pathway, IL-8 production and neutrophil recruitment during bacterial infection (81, 82). There is a novel mechanism of pathogen-host interaction in human airway epithelial cells and in mouse lungs mediated by sRNA-52320-induced attenuation of the immune response. It has been suggested that in immunocompromised individuals, sRNA-52320 enables *P. aeruginosa* to establish chronic lung infection (81).

#### 3.3.2 OMVs connect periodontal disease and inflammatory respiratory diseases *in vitro*

OMVs derived from *P. gingivalis*, a periodontal pathogen, disrupted the distribution of tight junction proteins resulting in disrupting the intact lung epithelial barrier system (7) (**Figure 1**). OMVs were also shown to significantly induce cell apoptosis, decreasing the viability of lung epithelial cells due to prominent cytotoxic effects and leading to pulmonary inflammation (6). As the gingipains of *P. gingivalis* have been reported to be involved in aspiration pneumonia and gingipains are important components of *P. gingivalis* OMVs, the gingipains of *P. gingivalis* OMVs may be a critical factor in *P. gingivalis* OMVs induced pulmonary inflammation (83). This *in vitro* experiment was the first to show that *P. gingivalis* OMVs participated in pulmonary pathology, suggesting a close relationship between periodontal disease and inflammatory respiratory diseases. Further verification of the relationship between *P. gingivalis* OMVs and pulmonary inflammation, as well as the pathogenic pathway in animal models of periodontitis, may provide additional evidence.

#### 3.3.3 OMVs cause inflammation in human lung tissues

Rodent or cellular infection models differ from humans or lack tissue complexity. Jager J. et al. established and used a new infection model of *Legionella pneumophila* (*L. pneumophila*) infection in human lung tissue (84). The researchers demonstrated that *L. pneumophila* OMVs colocalized with alveolar macrophages and induced histological tissue destruction, which was similar to the damage caused by bacterial cells themselves in terms of quality and quantity (84). Loose extracellular matrix, protein exudation, and epithelial cell delamination from the connective tissue into the alveolar compartments were frequently observed (84). In summary, this experiment characterized early steps in human infection. It would be beneficial to understand the role of bacterial OMVs and their relationship with bacteria in inflammatory diseases if we could determine a practical concentration of OMVs during infection.

### 3.4 OMVs in sepsis-associated inflammation

Sepsis is a serious complication of infectious diseases characterized by life-threatening multisystem organ failure (85). Inflammation and intravascular coagulation are the primary factors in the etiology of sepsis (86). Host cells prevent bacteria and OMVs from spreading systemically through complicated crosstalk and interactions. An excessive immune response leads to systemic inflammation, blood flow changes and organ failure (87).

#### 3.4.1 OMVs induce inflammation and coagulation in endothelial cell models

Previous research was limited to the OMVs of pathogenic *E. coli*, while later studies showed that both nonpathogenic *E. coli* and pathogenic *E. coli* OMVs could initiate inflammatory cascades in human umbilical vein endothelial cells (HUVECs) (**Figure 1**) (8, 88). Endothelial cell stimulation by OMVs was shown to induce cytokine secretion, adhesion protein expression, and the presentation of the adhesion molecules E-selectin and P-selectin, which facilitate the migration of inflammatory cells to infection sites (88, 89). OMVs also upregulate tissue factor expression and downregulate thrombomodulin expression to shift the intravascular equilibrium toward coagulation. Stimulated HUVECs promoted the activation of platelets and the formation of prothrombotic monocyte-platelet aggregates (MPAs), thus increasing thrombus formation (8, 88, 90). Although HUVECs are the best characterized endothelial cells, there are still some limitations. Endothelial cells from different vascular beds obviously differ in their sensitivity to OMVs, as well as in their activation of the inflammatory response and coagulation cascade (91). Further investigation is required to determine the effect of OMVs on endothelial cells from other vascular beds.

#### 3.4.2 OMVs mediate inflammation and coagulation cascades in animal models

Sepsis triggered by OMVs mediates inflammation and coagulation cascades in an animal model (92, 93) (**Figure 1**). Mice subjected to cecal ligation and puncture to establish the sepsis model had *E. coli* and OMVs in their peritoneal fluid (94). *E. coli* OMVs infused via the jugular route elicit histological, physiological, and molecular changes in rats that are consistent with sepsis (92). *E. coli* OMVs injected intraperitoneally also induced the host systemic inflammatory response. This result is similar to clinically related symptoms of sepsis that are characterized by hypothermia, tachypnea, leukopenia, dysfunction of the lungs, disseminated intravascular coagulation, and the induction of IL-6 and TNF-α (93). However, compared with intraperitoneal injection of OMVs, LPS alone showed less lethality. Some recent studies suggested that treating OMVs with the LPS inhibitor polymyxin B leads to a reduction in OMV-induced lethality (93). In addition, intraperitoneal injection of OMVs resulted in decreased lethality in CD14^-/-^ mice compared with wild-type mice. Therefore, OMVs are more powerful inducers of sepsis than LPS alone. The role of OMV components in sepsis is still unknown. OMV cargoes other than LPS may be important in the pathogenesis of sepsis-related lethality (93).

### 3.5 OMVs in inflammation-related tumors

Chronic tissue inflammation caused by bacteria may facilitate tumor formation and development. Some recent studies pointed that OMVs also play a vital role in oral squamous cell carcinomas, gastric adenocarcinoma and colorectal cancer (9, 95, 96). Virulence factors delivered by bacteria OMVs are able to induce genomic damage in cancer cell line (97). Increasing evidence suggests that OMVs may repress translation and promote transcript decay of specific mRNAs by delivering differentially packaged sRNAs. OMVs may also effect immunomodulation in the tumor microenvironment by directly binding to immune cells. A recent study indicated that sRNA-23392 packaged by *P. gingivalis* OMVs promote the invasion and migration of oral squamous cell carcinoma cells by targeting desmocollin-2 (9) (**Figure 1**). SRNA-2509025 and sRNA-989262 packaged by *H. pylori* OMVs attenuate IL-8 production in AGS cells (95). After binding to Siglic-7 (sialic acid-binding immunoglobulin-like lectin) of dendritic cells, *F. nucleatum* OMVs inhibit dendritic cell activation, which allow the tumor to evade immune surveillance in colorectal cancer (96) (**Figure 1**).

These studies provide a novel approach for understanding the connection between OMVs-related inflammation and tumors and also open a novel dimension for mediating tumor immune modulation by bacteria OMVs. Initial evidence that blocking the interaction between bacteria OMVs and immune cells may represent a potential strategy for alleviating the progression of bacteria-associated tumors. However, further investigations are expected to verify the mechanisms of OMVs in animal experiments and not only cell lines. The role and mechanisms of OMVs in inflammation-related tumors need to be demystified in the future.

## 4 The mechanisms of OMVs in inflammatory signal cascades

### 4.1 OMVs recognized by pattern recognition receptors on the cell membrane

**Figure 2:**
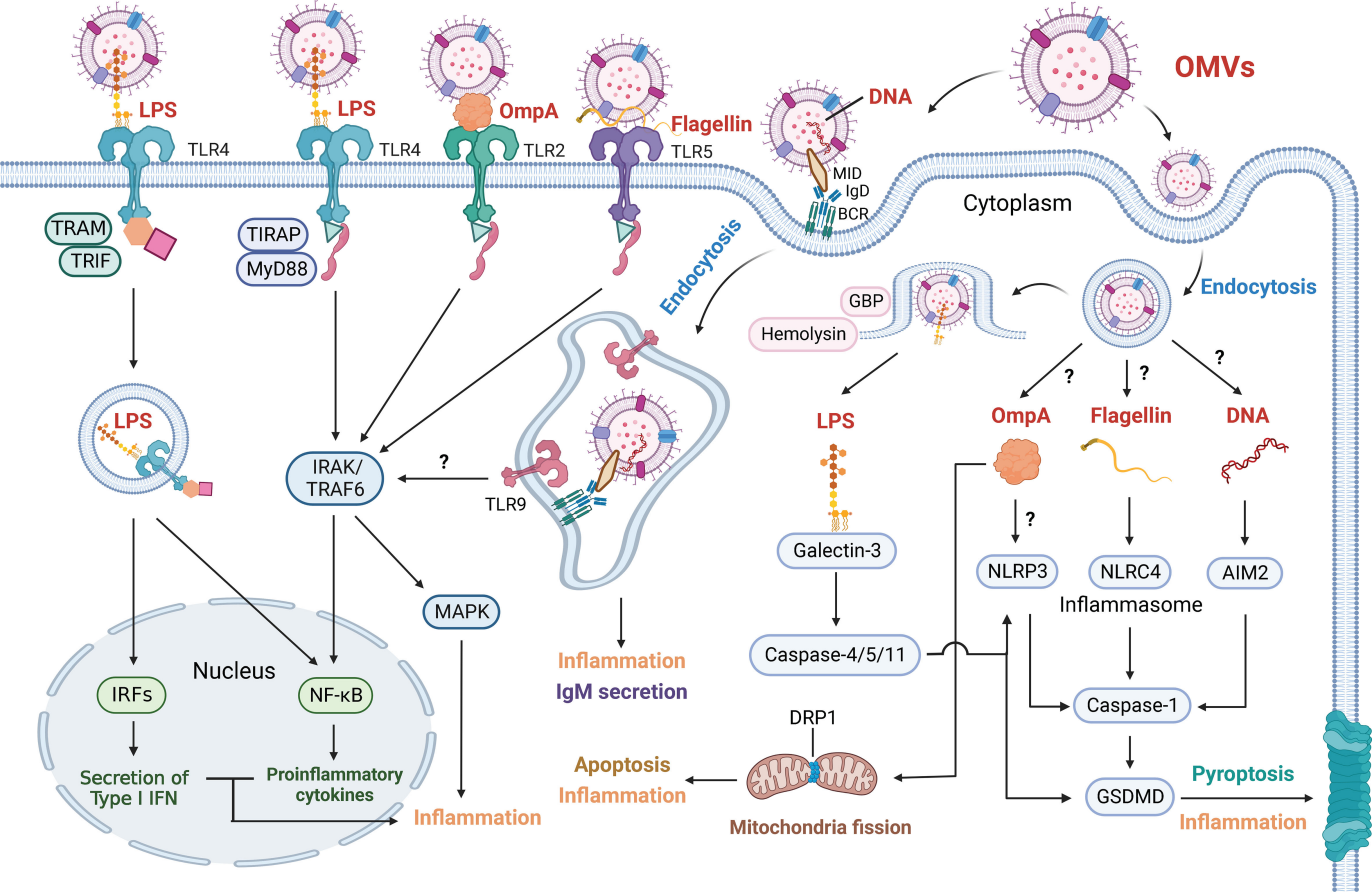
Outer Membrane Vesicles Induce Various Inflammatory Response. OMV-associated cargoes (red font), including LPS, OmpA, flagellin and DNA, can elicit various inflammatory responses. These factors are directly detected by cell membrane receptors or enter target cells via endocytosis and are detected by cell-cytoplasm receptors. OMV-derived LPS is sensed by TLR4. The LPS-TLR4 complex then recruits TIRAP and MyD88 to activate IRAK and TRAF6, which stimulates the NF-κB and MAPK signaling pathways. The LPS-TLR4 complex can also be internalized and recruit TRAM and TRIF, resulting in the NF-κB signaling pathway and the secretion of type I IFN via IRFs. GBP and hemolysin can induce the rupture of OMV-containing vacuoles and increase LPS exposure to cytosolic sensors. Galectin-3 forms ternary complexes with LPS and caspase-11, thereby amplifying caspase-4/11 oligomerization and exacerbating pyroptosis. Activated caspase-4/5/11 proteolytically triggers GSDMD pore formation to induce pyroptosis and inflammation. Activated caspase-4/5/11 also triggers the NLRP3 inflammasome, and caspase-1 is similarly activated to induce pyroptosis and inflammation. OMV-derived OmpA is not only sensed by TLR2 to induce the NF-κB and MAPK signaling pathways but also activates the NLRP3 inflammasome. In addition, OmpA stimulates the host GTPase DRP1 to promote its accumulation on mitochondria, which results in apoptosis and inflammation. OMV-derived flagellin is sensed by TLR5 and induces the NF-κB and MAPK signaling pathways. Cytosolic flagellin activates the NLRC4 inflammasome, leading to caspase-1-mediated pyroptosis and inflammation. OMVs containing MID mediate contact with the IgD BCR, resulting in endosome formation via endocytosis. Subsequently, TLR9 is recruited and senses DNA in endosomes, contributing to inflammation and the secretion of IgM. OMV-derived DNA is also sensed by the AIM2 inflammasome. Whether OMVs mediate NF-κB and MAPK signaling via TLR9 has not been shown. How OMV-containing vacuoles release OmpA, flagellin and DNA and how OmpA activates NLRP3 inflammasomes also remain unclear.

A variety of pattern recognition receptors (PRRs) on the membrane of immune cells and nonimmune cells can recognize the pathogen-associated molecular patterns (PAMPs) of OMVs, triggering a rapid inflammatory response that is critical to innate immunity. Among them, LPS is recognized by TLR4, peptidoglycan, lipopeptide/proteins, outer membrane protein FomA and OmpA are recognized by TLR2, and flagellin is recognized by TLR5 (**Figure 2**).

#### 4.1.1 LPS is recognized by TLR4

LPS is an important virulence cargo in bacterial OMVs, such as those of *E. coli*, EHEC, *B. fragilis* and *Salmonella typhimurium* (*S. typhimurium*), that is recognized by TLR4 (16, 98–101) (**Table 1**). Initially, LPS-binding protein (LBP) binds LPS and extracts and transfers lipid A molecules from LPS to cluster of differentiation 14 (CD14). Interactions among LPS, LBP and CD14 contribute to the remodeling and presentation of lipid A of LPS to myeloid differentiation factor 2 (MD-2) (117, 118). Subsequently, MD-2 modularizes the lipid A moiety with a sandwich-like structure, which then binds to TLR4 to induce dimerization (119). After binding to the LPS/MD-2 complex, TLR4 can initiate two different signaling pathways.

**Table 1:** Examples of Inflammation Signal Cascades Activated by Outer Membrane Vesicles.

Bacteria Producing OMVs	OMVs-associated Cargo	Target Cell Sensors	Effect of Activation by OMVs	Reference
*E. coli*	LPS	TLR4-MyD88	Inflammation	(98)
		TLR4-TRIF	Inflammation	(98)
		NLRP3	Pyroptosis, Inflammation	(98, 102, 103)
		caspase-4/11	Pyroptosis, Inflammation	(98, 103, 104)
	?	Mitochondria	Apoptosis, Inflammation	(19)
*P. gingivalis*	PG	NOD1, NOD2	Inflammation	(105)
*P. gingivalis*	Lipopeptide/proteins	TLR2	Inflammation	(106)
	?	NLPR3	Pyroptosis, Inflammation	(107)
	?	AIM2	Pyroptosis, Inflammation	(107)
*T. denticola*	Lipopeptide/proteins	TLR2	Inflammation	(106)
	?	NLPR3	Pyroptosis, Inflammation	(107)
	?	AIM2	Pyroptosis, Inflammation	(107)
*T. forsythia*	Lipopeptide/proteins	TLR2	Inflammation	(106)
	?	NLPR3	Pyroptosis, Inflammation	(107)
	?	AIM2	Pyroptosis, Inflammation	(107)
EHEC	LPS	TLR4	Inflammation	(16)
	flagellin	TLR5-MyD88	Inflammation	(16, 108)
	hemolysin	Mitochondria	Apoptosis, Inflammation	(57, 109)
*F. nucleatum*	FomA	TLR2	Inflammation	(14)
*A.actinomycetemcomitans*	RNA	TLR8	Inflammation	(110)
	PG	NOD1, NOD2	Inflammation	(105)
*M. catarrhalis*	DNA	TLR9	Inflammation, IgM secretion	(111)
*H. pylori*	PG	NOD1	Inflammation	(112)
*P. aeruginosa*	?	NLPR3	Pyroptosis, Inflammation	(19, 113, 114)
	PG	NOD1	Inflammation	(112)
	flagellin	NLRC4	Pyroptosis, Inflammation	(102)
	LPS	caspase-5/11	Pyroptosis, Inflammation	(98, 114)
	?	Mitochondria	Apoptosis, Inflammation	(19)
*N. gonorrhoeae*	?	NLPR3	Pyroptosis, Inflammation	(19)
	PG	NOD1	Inflammation	(112)
	PorB	Mitochondria	Apoptosis, Inflammation	(115)
*V. cholerae*	PG	NOD1, NOD2	Inflammation	(113)
UPEC	?	NLPR3	Pyroptosis, Inflammation	(19)
*B. pertussis*	LPS	NLPR3	Pyroptosis, Inflammation	(116)
		caspase-11	Pyroptosis, Inflammation	(98, 116)
*S. typhimurium*	LPS	TLR4-MyD88/TRIF	Inflammation	(100)
	flagellin	NLRC4	Pyroptosis, Inflammation	(102)
	LPS	caspase-11	Pyroptosis, Inflammation	(98)
*E. tarda*	hemolysin-LPS	caspase-4/11	Pyroptosis, Inflammation	(19)
*S. flexneri*	LPS	caspase-11	Pyroptosis, Inflammation	(98)
*A. baumannii*	OmpA	TLR2	Inflammation	(15)
		NLPR3	Pyroptosis, Inflammation	(15)
		Mitochondria	Apoptosis, Inflammation	(15)
*B. fragilis*	PG	TLR2	Inflammation	(99)
	LPS	TLR4	Inflammation	(99)
	RNA	TLR7	Inflammation	(99)

?, unclear or not known.

##### 4.1.1.1 Two signaling pathways downstream of LPS/MD-2/TLR4

For the first signaling pathway, surface dimerization of the TLR4/MD-2 complex recruits the adaptor proteins myeloid differentiation primary response 88 (MyD88) and Toll-interleukin I receptor domain-containing adaptor protein (TIRAP, a bridging adaptor protein between TLR4 and MyD88). MyD88 activates downstream IL-1 receptor-associated kinase (IRAK) and TNF receptor-associated factor 6 (TRAF6), subsequently stimulating the inflammatory response through the NF-κB and MAPK signaling pathways and the production of IL-6, IL-1β, IL-8 and TNF-α (10, 120–122).

In terms of the second signaling pathway, the dimerized TLR4/MD-2 complex can be internalized and then recruit TIR domain-containing adaptor protein inducing TRIF-related adaptor molecule (TRAM) and TRIF, which activate the NF-κB signaling pathway and induce the secretion of IFN via transcription factor interferon regulatory protein 3 (IRF3) (123, 124). Collectively, LBP and CD14 may act upstream of TLR4/MD-2 to engage and dissociate the lipid A molecule from LPS in OMVs, inducing the dimerization of TLR4 and activation of the NF-κB and MAPK signaling pathways.

#### 4.1.2 Peptidoglycan, lipopeptide/proteins, FomA and OmpA are recognized by TLR2

Peptidoglycan, lipopeptide/proteins, FomA and OmpA are recognized by TLR2 on the cell membrane (**Table 1**). Similar to the activation of TLR4, after binding to PAMPs, TLR2 recruits MyD88 to stimulate the NF-κB and MAPK signaling pathways (125). Early studies showed that PG is recognized by TLR2 (99, 126) (**Table 1**). A experiment that showed purified PG can activate a TLR2 reporter cell line excluded the effect of the contamination of lipoproteins and lipoteichoic acids observed in previous studies (127). However, PG-induced inflammatory responses could not be completely suppressed and low levels of IL-6 and TNF-α could be detected with the TLR2 antibody treatment, which indicated that other sensors are involved in PG-induced inflammatory responses, and these sensors are NOD1 and NOD2, as mentioned previously and will be elaborated later (128). In addition, the lipopeptide/proteins on periodontal pathogen OMVs can activate TLR2, and their immunogenicity depends on the length of their alkyl chain. Chain lengths of C18, C16, and C14 reduce the stimulation of TLR2 in sequence (11). Consistently, when OMVs are standardized by protein concentration, the stimulation of TLR2 by different periodontal pathogen OMVs did not differ (106). FomA of *F. nucleatum* OMVs was shown to trigger immunity in IECs by inducing NF-κB activation in a TLR2-dependent manner (14). The researchers suggested the involvement of the TLR2/TLR2 homodimer but not the TLR1/TLR2 heterodimer or the TLR2/TLR6 heterodimer (14). In addition, OmpA of *A. baumannii* OMVs also activates an intense proinflammatory response via the TLR2, NF-κB and MAPK signaling pathways (15).

A recent report demonstrated that active *P. gingivalis* peptidylarginine deaminase (PPAD) can citrullinate cell surface proteins of *P. gingivalis* that function as TLR2 ligands (129) (**Table 1**). A large amount of active PPAD on *P. gingivalis* OMVs is required for the activation of TLR2 to stimulate the inflammatory response (129). Furthermore, fimbriae from wild-type *P. gingivalis* strains, but not PPAD-deficient *P. gingivalis* strains, induced cytokine production via activation of the TLR2, NF-κB and MAPK signaling pathways (129). Despite the presence of PPAD in OMVs, there was no citrullination of fimbriae in wild-type *P. gingivalis* strains (130, 131). Notably, challenging techniques may be responsible for the lack of detection of citrullinated peptides, and strain-specific differences in citrullination efficiency are likely.

#### 4.1.3 Flagellin is recognized by TLR5

Flagellin is recognized by TLR5. H7 flagellin on EHEC OMVs was reported to trigger the inflammatory response through TLR5, leading to IL-8 production by human IECs (16) (**Table 1**). Accordingly, TLR5 uses its lateral side to interact with the three helices of the flagellin D1 domain (132). Two TLR5-flagellin 1:1 heterodimers assemble into a 2:2 tail-to-tail signaling complex (132). The flagellin-TLR5 complex induces the MyD88-NF-κB signaling pathway in epithelial cells, dendritic cells and monocytes, which stimulates the immune response against bacteria with flagellin (108, 133, 134).

### 4.2 OMVs recognized by pattern recognition receptors in the cytosol

In addition to being recognized by TLRs on the cell membrane, OMVs can also be recognized by TLRs and NLRs in the cytosol after being endocytosed (135).

#### 4.2.1 RNA is recognized by TLR7 or TLR8

RNA on bacterial OMVs is recognized by TLR7 or TLR8 in the endosomes (**Table 1**). As an endosomal single-stranded RNA (ssRNA)-sensing receptor, the TLR7 and TLR8 MyDDosome signaling complex activates the NF-κB signaling pathway and induces the nuclear translocation of IRF5/7, which promotes the secretion of IFN-β, IFN-γ, TNF-α and IL-12 (136, 137). Confocal microscopy showed that *B. fragilis* OMVs could transmit RNA to Caco-2 cells intracellularly (99). In contrast to unstimulated controls, stimulation with *B. fragilis* OMVs led to significant activation of TLR7 in HEK-Blue cells expressing TLR7 (99). However, neither *B. fragilis* OMVs nor *B. fragilis* cells activated TLR8. The recognition of RNA by TLRs is species specific. RNA from *Aggregatibacter actinomycetemcomitans* (*A. actinomycetemcomitans*) OMVs was shown to induce TLR8 and NF-κB signaling and stimulate TNF-α release by macrophages (110). *A. actinomycetemcomitans* OMVs delivering RNA can cross the blood-brain barrier (BBB) and promote TNF-α production, but whether they act via TLR8-dependent signaling is not clear (17, 110, 138). In addition, Streptococcus Group B RNA is recognized by TLR7, whereas RNA from *E. coli* is recognized by TLR8 (139). Evidence of different ligand sensitivities may provide an explanation for bacterial species specificity (140). These reports did not investigate RNA structure, and so about it is unclear whether RNA in OMVs is characterized by single strains.

#### 4.2.2 DNA is recognized by TLR9

DNA on bacterial OMVs is recognized by TLR9 in the endosomes (**Table 1**). DNA from periodontopathogenic bacteria, such as *A. actinomycetemcomitans*, *P. gingivalis*, and *Peptostreptococcus micros*, activate the production of TNF-α and IL-6 through TLR9 signaling in a dose-dependent manner (12) (**Table 1** and **Figure 2**). TLR9 is a DNA recognition receptor that mediates the MyD88 and NF-κB signaling pathways and type I IFN secretion (141). Upregulation of TLR9 was shown in OMV-stimulated B cells. *M. catarrhalis* OMVs containing the superantigen MID mediate contact with the IgD B-cell receptor (BCR) to form lipid rafts, resulting in cross-linking and endocytosis by B cells (111). Internalized antigen and BCR signaling recruit TLR9 to early endosomes, which allows TLR9 to recognize the antigen via its unmethylated CpG-DNA motif ligand (111). TLR9 signaling may contribute to nonspecific antibody production by reducing the threshold for B-cell activation (111). Overall, DNA in OMVs is sensed by TLR9 and mediates IL-6 and IgM secretion (111). Bacterial OMVs that mediate NF-κB signaling, MAPK signaling and type I IFN secretion via TLR9 signaling have not been reported. Whether DNA is located within or on the surface of vesicles remains controversial. A proportion of DNA that remained inaccessible to deoxyribonuclease showed that DNA may be located within vesicles (142). However, the presence of extracellular DNA in purified OMV preparations indicated that the DNA strands may be located on the vesicle surface, potentially linking the factors in a DNA/OMV network (143).

#### 4.2.3 Peptidoglycan is recognized by NLRs

After entering host cells, OMV-associated PG is recognized by NLRs in the cytosol, NOD-containing protein 1 (NOD1) and NOD-containing protein 2 (NOD2) (13, 144). Studies revealed that OMVs derived from *H. pylori*, *P. aeruginosa* and *Neisseria gonorrhoeae* (*N. gonorrhoeae*) enter the epithelium, induce NOD1-dependent responses and activate NF-κB signaling (112) (**Table 1**). *V. cholerae* OMVs interact with NOD1 and NOD2, thereby activating NF-κB signaling and IL-8 production in HEK293T cells and IL-8 production in THP1 and HeLa monocytes (113) (**Table 1**). The periodontal pathogens *A. actinomycetemcomitans* and *P. gingivalis* also produce OMVs that activate NOD1 and NOD2, resulting in activation of the NF-κB signaling pathway (105) (**Table 1**).

Specifically, NOD1 and NOD2 recognize the major components of PG D-glutamyl-mesodiaminopimelic acid (iE-DAP) and muramyl dipeptide (MDP), respectively (145, 146). Their assembly recruits RIPK2 through characterized by a caspase-recruitment domain (CARD)-CARD interactions, leading to the formation of the TAB1/TAK1 complex (147). TAK1 becomes activated and then stimulates the downstream IKK complex, resulting in IKK phosphorylation, activation of the NF-κB signaling pathway, and the secretion of IL-6 and IL-8 (148). NOD oligomerization also recruits the TAB1/TAK1 complex to activate the MAPK signaling pathway via the upstream activation of MKKs (149). In addition, the recognition of NOD1 and NOD2 binding to TNF receptor-associated factor 3 (TRAF3) induces TRAF-associated NF-κB activator-binding kinase 1 (TBK1)/IKKϵ activation and IRF transcription factor dimerization, resulting in type I IFN production (144, 150). Furthermore, NOD1 can recognize PG within internalized OMVs and then induce autophagy, resulting in the degradation of OMVs and cargo release (151).

However, the mechanism by which NOD1 and NOD2 detect OMV-associated cargoes remains unclear, and their exact intracellular location is unknown. Studies have demonstrated that NOD1/2 can be absorbed into the plasma membrane and endosomes due to a lack of transmembrane domains (152). These factors can be anchored to the plasma membrane via cytoskeletal components or membrane binding proteins or to the endosomal membrane by the proteins SLC15A3 or SLC15A4, which are necessary for signal transduction (153). Recent research has reported that membrane recruitment and immune signaling require NOD1/2 S-palmitoylation. S-palmitoylation mediated by the palmitoyltransferase ZDHHC5 is characterized by reversible localization and rapid changes in NOD1/2, which is essential for its response to PG and the establishment of effective immune responses to bacterial infection (153). However, NOD1/2 palmitoylation has not been examined during the recognition of OMV-associated PG.

### 4.3 OMVs activate the inflammasome

#### 4.3.1 OMVs activate canonical inflammasomes

In recent years, NLRP3, NLRC4 and AIM2 have been reported to participate in the formation of inflammasomes (18, 154). Inflammasomes are intracellular multimeric complexes that assemble in the cytosol of different immune cells and epithelial cells, particularly macrophages, and activate various receptors and caspases (154). After PAMPs are recognized by PRRs, the expression of inflammasome components is upregulated (155, 156). This is defined as canonical inflammasome activation. NLRP3, NLRC4 and AIM2 inflammasomes serve as platforms to engage and oligomerize pro-caspase-1 to form activated caspase-1 (18) (**Figure 2**). Activated caspase-1 converts pro-IL-1β and pro-IL-18 to their active forms (IL-1β and IL-18) and proteolytically stimulates gasdermin-D (GSDMD) (157, 158). Subsequently, the amino-terminal fragment of GSDMD assembles into membrane pores, and the release of IL-1β and IL-18 recruits immune cells to resist infection (159). Ultimately, the loss of cell membrane integrity induces pyroptosis (18).

##### 4.3.1.1 OMVs activate the NLRP3 inflammasome

Accumulating evidence has revealed that various bacteria, including *P. aeruginosa*, uropathogenic *E. coli* (UPEC), *N. gonorrheae*, *P. gingivalis*, *T. denticola*, *T. forsythia*, *Bordetella pertussis* (*B. pertussis*), *A. baumannii* and *E. coli*, produce OMVs that activate the NLRP3 inflammasome, resulting in pyroptosis and inflammation (15, 19, 101, 102, 107, 114, 116) (**Table 1**). The NLRP3 inflammasome is a multiprotein complex consisting of a sensor (NLRP3), an adaptor (ASC) and pro-caspase-1. Upon activation by PAMPs and DAMPs, NLRP3 oligomerizes and activates caspase-1 which proteolytic activates the proinflammatory cytokines IL-1β and IL-18. In mouse macrophages, TLR4 signaling via MyD88 reportedly initiates the NLRP3 inflammasome rapidly and non-transcriptionally by stimulating its deubiquitination (160). In comparison with *A. baumannii* OMVs, OmpA-deficient OMVs induced significantly reduced expression of NLRP3 (15). However, studies on the mechanism of NLRP3 inflammasome activation have not focused on OMV proinflammatory components. More investigations are needed to fully understand NLRP3 inflammasome activation by bacterial OMVs.

##### 4.3.1.2 OMVs activate the NLRC4 inflammasome

*P. aeruginosa* OMVs reportedly activate the NLRC4 inflammasome with flagellin protein (102) (**Table 1**). Robust activation of NLRC4 induced caspase-1 and the secretion of IL-1β after treatment with *S. typhimurium*-derived OMVs (102). Specifically, in response to engagement with the NLRC4 monomer, flagellin-bound neuronal apoptosis inhibitory protein 5 (NAIP5) is assembled into an activated oligomeric complex and directly recruits caspase-1, subsequently mediating pyroptosis (161–164). Consistently, *S. typhimurium* OMVs lacking flagellin failed to robustly activate the NLRC4 inflammasome (102). These findings suggest a potential role of OMV-associated flagellin in NLRC4 inflammasome activation.

##### 4.3.1.3 OMVs activate the AIM2 inflammasome

Two methods of DNA encapsulation were explored to demonstrate the origin of DNA within OMVs :(1) DNA existing in the periplasm and along with other periplasmic components, is encapsulated within an OMV, or (2) DNA existing in the extracellular environment is internalized within an OMV (165). OMVs from several gram-negative bacteria, such as *P. gingivalis*, *T. denticola*, and *T. forsythia*, may deliver DNA to the host cytosol and activate the AIM2 inflammasome (107) (**Table 1**). AIM2 is characterized by a C-terminal HIN domain that directly binds to dsDNA in the cytosol through electrostatic interactions during inflammasome assembly (166). The AIM2-associated N-terminal PYD domain is in charge of recruiting ASC (166). Scholars have suggested that the mechanism of ASC-PYD complex assembly and the polymerization of AIM2-PYD is similar to the mechanism through which actin nucleation factors initiate actin polymerization (166). However, how the dsDNA-bound HIN domain initiates the recruitment of ASC through the PYD domain has not been determined. As an intracellular dsDNA sensor, AIM2 forms an inflammasome with ASC to activate caspase-1 and the production of the inflammatory cytokines IL-1β and IL-18 (116, 167). The AIM2-ASC platform has also been shown to activate caspase-8, leading to apoptosis in caspase-1-deficient macrophages during *Francisella tularensis* infection (168). However, a lack of sequence specificity fails to distinguish self-DNA and microbial DNA.

Notably, the activation of canonical inflammasomes induces inflammatory responses, and pyroptosis provides host defense by disrupting the cellular environment (158). However, we cannot entirely rule out the possibility that other OMV cargoes may be potential signals that activate canonical inflammasomes.

#### 4.3.2 OMVs activate noncanonical inflammasomes

In contrast to canonical inflammasome activation, PRR signaling is not essential for noncanonical inflammasomes, which consist of pro-caspase-4 and -5 in humans or pro-caspase-11 in mice (169). These components can directly bind to LPS aggregates and induce oligomerization, resulting in inflammasome activation (170). In terms of the number and length of lipid chains in lipid A of LPS, caspase-4/5/11 can recognize a greater variety of lipid A variants than the TLR4/MD-2 complex (171). Activated caspase-4/5/11 proteolytically triggers GSDMD pore formation, which damages homeostasis and induces pyroptosis in the same manner as canonical inflammasomes do (170, 172, 173) (**Figure 2**). Activated caspase-4/5/11 stimulates the NLRP3 inflammasome to activate IL-1β and IL-18 production (98). It has been demonstrated that bacterial OMVs can activate noncanonical inflammasomes, including *E. coli*, *Edwardsiella tarda* (*E. tarda*), *P. aeruginosa*, *N. gonorrheae*, *Shigella flexneri* (*S. flexneri*), *S. typhimurium*, *B. pertussis* and *A. baumannii* (15, 19, 98, 104, 114, 116) (**Table 1**). But free/purified LPS may be recognized differently to OMV-associated LPS. Previous study demonstrated that free/purified LPS added to the macrophage culture does not enter the cytosol to trigger caspase-11-dependent response compared to OMV-associated LPS (103).

##### 4.3.2.1 Factors that affect LPS-induced activation of noncanonical inflammasomes

Type I IFN induced by TLR4-TRIF axis signaling is required for OMV-induced activation of noncanonical inflammasomes and enhances guanylate binding protein (GBP) expression (98). Various GBPs, such as GBP1, GBP2 and GBP5, bind cytosolic OMVs by direct LPS-protein interactions, inducing endosomal lysis or LPS release and promoting the activation of noncanonical inflammasome-dependent pyroptosis (98, 174). However, GBP-chr3-deficient cells that were treated with OMVs contained the same levels of cytosolic LPS as wild-type cells, which indicated that GBPs may indirectly participate in endosomal lysis and LPS release (103). Therefore, it is possible that GBPs act as platforms for caspase-4/5/11 activation. In addition, a study reported that the overexpression of hemolysin activated IL-18 production in a caspase-11-dependent manner in intestinal inflammation (104). Hemolysin can induce the rupture of OMVs and promote LPS exposure to cytosolic sensors during noncanonical inflammasome activation (104).

In macrophages treated with OMVs, galectin-3, a nucleocytoplasmic protein, has been reported to promote the activation of noncanonical inflammasomes by binding to cytoplasmic LPS glycans (175). Colocalization of galectin-3, LPS and caspase-11 was revealed by immunofluorescence staining. Galectin-3 uses the carbohydrate recognition domain (CRD) to assemble ternary complexes with LPS and caspase-11 in a carbohydrate recognition-dependent manner, thereby amplifying caspase-11 oligomerization and exacerbating pyroptosis (175). LPS from OMVs of different bacterial species interacts with different domains of caspase-11. The activated caspase-11 and recruitment domain (CARD) coimmunoprecipitated with galectin-3 in the presence of *Salmonella minnesota* LPS and *E. coli* LPS (175). These studies provided insights into the manipulation of noncanonical inflammasome signaling by restricting bacterial colonization. Therefore, identifying the host factors that affect LPS-induced activation of noncanonical inflammasomes may help promote OMV-mediated pyroptosis activation to prevent infection.

### 4.4 OMVs induce mitochondrial dysfunction

Mitochondria maintain intracellular homeostasis by modulating metabolism, immunity and apoptosis (176). Many pathogens have evolved to deliver virulence factors that target mitochondria and interfere with cell defense (177). This also explains why pathogen OMVs can induce macrophage death and IL-1β secretion, although they can evade TLR4 and caspase-11 detection by modifying LPS (20). Recent research has shown that macrophages exposed to OMVs from *N. gonorrheae*, UPEC and *P. aeruginosa* induce mitochondrial dysfunction (19) (**Table 1**). Specifically, this treatment triggered cytochrome C release, decreased the mitochondrial membrane potential, and depleted the unstable B-cell lymphoma 2 (BCL-2) family member myeloid cell leukemia 1 (MCL-1), which inhibited the synthesis of host proteins, leading to BCL-2-associated x protein (BAX)-dependent mitochondrial apoptosis (19, 178, 179). It is worth mentioning that mitochondrial apoptosis and potassium ion efflux can activate the NLRP3 inflammasome, triggering pyroptosis and inflammation after OMV exposure *in vitro* (180, 181). However, the specific components involved in mitochondrial dysfunction have not been identified.

OmpA, PorB and hemolysin were demonstrated to induce cell death and inflammation. In a mouse model of lung infection, *A. baumannii* used OMVs to promote bacterial dissemination and pathogenesis (182). OMVs containing OmpA are absorbed by cells to stimulate the host cell GTPase dynamin-related protein 1 (DRP1). DRP1 is activated by OmpA and accumulates in mitochondria, which results in the production of ROS, mitochondrial fragmentation, and cell death (182) (**Figure 2**). Additionally, direct insertion of the porin into mitochondria could be a potential mechanism by which OMV-OmpA induces mitochondrial fragmentation, which leads to cytochrome C release, thus damaging mitochondrial function (182). PorB from *N. gonorrheae* OMVs colocalized with Tom20, the translocase on the outer mitochondrial membrane, which influenced mitochondrial membrane potential, activated cytochrome C secretion, and subsequently induced apoptosis (115). However, the mechanisms of PorB translocation into mitochondria remain unclear. There are currently three ideas to explain this mechanism. First, PorB separates from cytosolic OMVs and translocates into mitochondria via host import machinery (183). Second, OMVs directly interact with mitochondria to deliver PorB (115). In addition, a close association between PorB and isolated mitochondria-enriched fractions may transfer proteins to mitochondria or promote membrane fusion (184). Interestingly, PorB may also target the mitochondrial inner membrane to directly initiate a decrease in membrane potential (185). Furthermore, when OMVs enter endolysosomal compartments, EHEC-hemolysin can dissociate from OMVs, escape from endolysosomes through pore-form activity and target mitochondria after endosomal acidification, which also contributes to mitochondrial dysfunction and cell death (57, 109). Notably, the dissociation of EHEC-hemolysin from endolysosomes to mitochondria may be related to a putative mitochondrial targeting signal in the N-terminal region of the EHEC-hemolysin sequence but not require OMVs (186). However, the mechanisms of the interaction of EHEC-hemolysin with mitochondria remain unclear.

Intriguingly, mitochondrial dysfunction mediated by *P. gingivalis* OMVs activated the release of inflammatory cytokines and promoted glycolysis in macrophages, leading to cell death (46, 187). Subsequently, inflammatory cytokines are released into the extracellular environment and perpetuate local inflammation, which enables the return of nutrients to *P. gingivalis* and other plaque bacteria through gingival exudates (188). Therefore, further study on the mechanism that regulates this metabolic transfer may aid in the discovery of new inflammatory response mechanisms.

## 5 OMVs affect inflammation in distant organs or tissues

### 5.1 Atherosclerosis

AS, which is a chronic inflammatory disease, is characterized by cholesterol accumulation, foam cell formation and macrophage recruitment to the arterial wall (21). The accumulation of low-density lipoprotein (LDL) is considered a crucial risk factor for AS, but native LDL is not transported into macrophages (189). After being modified by oxidation or aggregation, decorated LDL tends to be absorbed by macrophages and is responsible for foam cell formation (190). Studies in the past decade have examined the role of infection in AS pathogenesis. Bacterial infection in the oral and gastrointestinal tract induces endothelial cell damage, which is related to AS pathogenesis (191, 192). However, only *P. gingivalis* OMVs and *H. pylori* OMVs were shown to be involved in AS (**Figure 1**).

#### 5.1.1 *P. gingivalis* OMVs are involved in AS

Patients with periodontal disease tend to experience transient bacteremia due to mechanical friction or iatrogenic injury, and OMVs released from periodontal pathogens advance into deeper epithelial layers and the bloodstream (193, 194). Studies have reported that bacteria in the oral microbiome, such as *P. gingivalis*, *T. forsythia*, *T. denticola*, *A. actinomycetemcomitans*, and *Prevotella intermedia*, are present in atherosclerotic plaques obtained from the carotid and coronary arteries of patients (195). Studies also have provided evidence that *P. gingivalis* OMVs are involved in AS. In the presence of LDL, low concentrations of *P. gingivalis* OMVs induce the formation of foam cells. *P. gingivalis* OMVs promoted LDL absorption and modification by macrophages (196). OMVs have been shown to use gingipains to aggregate and transfer LDL via apoB-100 proteolysis to stimulate foam cell formation (196, 197). In addition, *P. gingivalis* OMVs promoted calcification in vascular smooth muscle cells, further indicating their involvement in AS. These factors upregulated the expression of classical markers of osteoblastic differentiation and mineralization in vascular smooth muscle cells *in vitro* and ex vivo through ERK1/2-Runx2 signaling (198). Due to high invasive abilities, *P. gingivalis* OMVs induced the expression of CXCL8 and E-selectin in endothelial cells, which significantly initiated monocyte adhesion to HUVECs, consequently resulting in atherosclerotic plaques (199). However, there are still some limitations. More research is required to investigate whether bacteria or OMV components participate in LDL aggregation and transfer and how *P. gingivalis* OMVs participate in AS pathogenesis in addition to LDL surface modification. It is still unclear what the actual levels of bacterial OMVs are in the circulation following bacteremia.

#### 5.1.2 *H. pylori* OMVs are involved in AS

*H. pylori*-specific DNA and the virulence factor CagA are present in atherosclerotic plaques obtained from the coronary arteries of patients (200). It has been reported that treatment with *H. pylori* OMVs increased cholesterol levels, promoted apoptosis in the arterial lumen and accelerated coronary artery atherosclerotic plaque formation in ApoE^-/-^ mice (21). *In vitro*, *H. pylori* OMVs promoted apoptosis and suppressed the proliferation of HUVECs in a concentration-dependent manner (21). Specifically, CagA and LPS from OMVs affected these processes via the ROS/NF-κB signaling pathway and mediated the release of IL-6 and TNF-α (21). Here, we provide novel insight into how target cells may be induced to release CagA-containing extracellular vesicles that are involved in AS due to *H. pylori* OMVs delivering virulence components (such as CagA) to the epithelium (201). *H. pylori* OMVs can increase epithelial permeability, thereby crossing the compacted epithelium monolayer, although there is a lack of evidence on whether or how *H. pylori* OMVs access the blood circulation (202). More fundamental research should be conducted to clarify the mechanism and virulence components of *H. pylori* OMVs in AS. Some damage mediated by *H. pylori* in atherosclerotic plaques is direct, and OMVs derived from *H. pylori* can be transferred to the plaques, promoting AS (203). It should be noted that OMVs may be by in atherosclerotic plaques and function locally, although this remains to be validated.

### 5.2 Alzheimer’s disease

AD is a neuroinflammatory disease with a long duration and generally slow progression. Neuroinflammation, which is the main pathological factor of AD, is characterized by activated glial cells such as astrocytes and microglia and proinflammatory cytokine production (204). The hallmark pathology of AD includes extracellular amyloid (Aβ) plaques and phospho-tau bound to neurofibrillary tangles (NFTs) (205). Specifically, increased permeability of the BBB is one of the requirements for infectious stimulation in AD pathogenesis (206). It has been shown that the function of the BBB relies on the tight junctions between capillary endothelial cells, and claudin-5 is an important protein in tight junctions (207). The glycogen synthase kinase 3β (GSK-3β) pathway also plays a crucial role in cognitive dysfunction associated with AD by inducing neuroinflammation and tau hyperphosphorylation (208). Previous results have shown that an abnormal gut microbiota (GM) OMVs and periodontal pathogens OMVs are closely associated with AD (22, 209) (**Figure 1**). Notably, OMV-associated LPS may be more toxic than free/purified LPS. Even in settings of increased BBB permeability, free/purified LPS only minimally crosses the BBB (210). Yet, OMVs can cross the BBB and are enriched with LPS causing peripheral and central nervous system inflammation (210). OMVs may represent an important alternate pathway of LPS brain entry. Collectively, OMVs might become more important in enabling transport of bacterial virulence factors.

#### 5.2.1 GM-derived OMVs are involved in AD

Recently, a study showed that after oral gavage of *E. coli*, *E. coli* OMVs were detected in the brain, but *E. coli* was not (211). Therefore, as a carrier for long-distance cargo transport, OMVs alter BBB permeability or penetrate the BBB in AD. For example, mice were injected with GM-derived OMVs from patients with AD into the tail vein, and they exhibited decreased claudin-5 expression and increased BBB permeability. This treatment activated astrocytes and microglia, increased the secretion of NF-κB, IL-1β, and TNF-α, and induced tau phosphorylation by stimulating GSK-3β (209). Overall, GM-derived OMVs trigger the GSK-3β signaling pathway, enhance tau phosphorylation, induce neuroinflammation and contribute to cognitive impairment (209). Previous studies have identified a significant positive association between *H. pylori* infection and AD development (212–214). The inflammatory response induced by *H. pylori* OMVs may further accelerate the development of *H. pylori* infection (215). A recent study first revealed role of *H. pylori* OMVs in AD development and progression (216). The authors indicated that *H. pylori* OMVs can reach the brain and be taken up by astrocytes (216). *H. pylori* OMVs exacerbate Aβ pathology and induce cognitive impairment via activation of glial cells and neuronal dysfunction. Upon the presence of H. pylori OMVs challenge, the complement component 3 (C3)-C3a receptor (C3aR) signaling played a critical role in mediating the interactions of astrocyte-microglia-neuron (216). These findings identify a mechanism based on initial evidence that pharmacological inhibition of GSK-3β signaling and C3-C3aR signaling prevents OMV induced neuroinflammation, activation of glial cells and neuronal loss and thereby rescues Aβ pathology and cognitive functions.

#### 5.2.2 Periodontal pathogen-derived OMVs are involved in AD

*P. gingivalis* OMV-delivered gingipains were involved in increased permeability of the human cerebral microvascular endothelial cell monolayer by degrading the tight junction proteins (occludin and Zonula occludens-1 (ZO-1)) responsible for BBB leakage *in vitro* (217, 218). LPS from *P. gingivalis* OMVs activates glial cells to induce neuroinflammation and the expression of NFTs and the AD marker protein Aβ (219). *In vivo*, three days after oral gavage, *P. gingivalis* OMVs could be detected in the cortex and hippocampus by confocal microscopy. Similarly, *P. gingivalis* OMVs administered to mice have been shown to activate glial cells, trigger NLRP3 inflammasomes, and promote tau phosphorylation, neuroinflammation, and memory dysfunction (22). In addition, after intracardiac injection, *A. actinomycetemcomitans* OMVs were able to pass through the BBB, induce neuroinflammation and promote the secretion of TNF-α via the TLR-8/NF-κB signaling pathway (110). A recent study demonstrated that OMVs could successfully deliver RNA into brain monocytes, resulting in the activation of IL-6 and neuroinflammation, suggesting a novel pathogenic mechanism in AD (17).

Additionally, as described by the polymicrobial synergy and dysbiosis theory, proinflammatory microbe-host communication could be induced by the remodified microbiota, rather than a single bacterial species (220). In fact, studies have demonstrated that OMVs extracted from the microbiota rather than a single strain in AD patients induced pathological changes (209). Thus, we need to be aware that periodontal pathogens and the GM as a whole may be involved in the pathogenesis of AD. Investigating OMVs from different sources and further identifying different substances in AD pathogenesis would yield some enlightening findings.

## 6 Conclusions

Bacterial OMVs are continuously secreted by gram-negative bacteria (35). Various pathogenic factors in OMVs and their ability to transfer toxic components to host cells and translocate into systemic circulation suggest that OMVs may play vital roles in bacterial pathogenesis by functioning as vehicles to deliver virulence cargoes (221). In this review, we examined the role of bacterial OMVs in inflammatory diseases, the mechanisms by which OMVs participate in inflammatory cascades, and the effects of OMVs on inflammation in distant organs or tissues.

In periodontal disease, bacterial OMVs participate in oral microorganism aggregation and invasion (42). The toxins and virulence factors delivered by gastrointestinal pathogenetic bacterial OMVs induce the destruction of the mucin barrier and bacterial colonization, resulting in gastrointestinal inflammation, while gastrointestinal commensal bacterial OMVs can mediate anti-inflammatory and barrier protection (54, 60, 62). Microorganisms also use OMVs to invade the lung epithelial barrier *in vivo* and *in vitro* (74, 78). Bacterial OMVs have been reported to trigger inflammation and coagulation cascades in sepsis (79, 92). Conclusively, OMVs can exert direct effects on cytotoxicity, apoptosis, or pyroptosis or indirectly target cells to produce tissue damaging components or cytokines. By understanding OMVs in inflammatory diseases, this knowledge is gradually being translated into novel ideas of OMV vaccines against inflammatory diseases.

Various PAMPs on OMVs are recognized by PRRs on cell membranes and in the cytosol, activate canonical inflammasomes and noncanonical inflammasomes, and induce mitochondrial dysfunction during the inflammatory cascade response. Due to their complexity and heterogeneity, OMVs may activate multiple receptors and mechanisms, such as LPS, OmpA, flagellin and DNA. However, we could not prove which mechanism is likely to be the principle method of OMV-induced inflammation, rather than functionally redundant mechanisms. Further understanding of the mechanism by which OMV cargoes initiate inflammatory cascades will promote the development of medicines to limit OMV-mediated inflammation. These findings regarding anti-inflammatory factors also provide new insights into their role in bacterial OMV assembly and expand our understanding of anti-inflammatory factor-containing OMVs as delivery vesicles in chronic inflammatory diseases.

In distant organ or tissue inflammation, *P. gingivalis* OMVs and *H. pylori* OMVs have been reported to be involved in atherosclerotic plaques (21, 199). Periodontal pathogen OMVs and GM OMVs play roles in neuroinflammation in AD (209, 219). These findings not only open a new dimension supporting the bacterial infection hypothesis underlying AS and AD pathogenesis but also identify OMVs as important players in the gut-brain axis. These reports demonstrated the potent long-distance cargo transport capacity of OMVs and provide new insights into the associations of multiple inflammatory diseases. However, existing reports have not provided direct evidence that OMVs in specific disease models are involved in pathogenesis in distant organs or tissues, and it is difficult to directly determine the relationship between periodontal disease or gastrointestinal inflammation and AS or AD. In detail, evidence of whether or how OMVs enter the blood circulation and cross the BBB and specific virulence factors in OMVs will clarify this mechanism.

In summary, the analysis of the role of OMVs in inflammatory diseases mainly concentrated on chronic inflammatory diseases in previous studies. However, the findings related to OMVs in autoimmune diseases except IBD have not been verified, and animal experiments exploring the role of OMVs in inflammation-related tumors have not been carried out. Notably, due to OMVs inheriting PAMPs of native bacteria, OMVs are effectively recognized, internalized and delivered by neutrophils to the target (222). Interestingly, bacterial OMVs can induce host mammalian cells to release stimulatory extracellular vesicles that enhance inflammatory responses (70). Numerous studies have indicated that OMVs act as more potent inducers of the inflammatory response. Based on knowledge of the source, biogenesis, structure and components of OMVs, it is reasonable to believe that OMVs may play a vital role in all inflammatory diseases through known or unknown mechanisms. Recent meta-analyses also identified that bacterial infection influence the development of central nervous system disease, psychiatric disorders, metabolic disease, autoimmune disease, etc. (212, 223–226). Further examination is needed to expand the existing limited understanding of the relationship between OMVs and inflammatory diseases and focus on the role and mechanism of OMVs in distant organ or tissue inflammatory pathology. The fundamental and clinical transformation of OMVs and inflammatory mechanisms should be strengthened.

## Author contributions

Conceptualization: SC, QL, XZ, and DM; writing-original draft preparation: SC; designing-figures and table: SC, QL, and XZ; writing-review and editing: SC and DM; supervision: SC, QL, XZ, and DM; funding acquisition: DM. All authors contributed to the article and approved the submitted version.
